# Depth-dependent attenuation and backscattering characterization of optical coherence tomography by stationary iterative method

**DOI:** 10.1117/1.JBO.28.8.085002

**Published:** 2023-08-24

**Authors:** Yaning Wang, Shuwen Wei, Jin U. Kang

**Affiliations:** Johns Hopkins University, Department of Electrical and Computer Engineering, Baltimore, Maryland, United States

**Keywords:** attenuation coefficient, optical coherence tomography, backscattering fraction, single scattering, multiple scattering, attenuation compensation

## Abstract

**Significance:**

Extracting optical properties of tissue [e.g., the attenuation coefficient (μ) and the backscattering fraction] from the optical coherence tomography (OCT) images is a valuable tool for parametric imaging and related diagnostic applications. Previous attenuation estimation models depend on the assumption of the uniformity of the backscattering fraction (R) within layers or whole samples, which does not accurately represent real-world conditions.

**Aim:**

Our aim is to develop a robust and accurate model that calculates depth-wise values of attenuation and backscattering fractions simultaneously from OCT signals. Furthermore, we aim to develop an attenuation compensation model for OCT images that utilizes the optical properties we obtained to improve the visual representation of tissues.

**Approach:**

Using the stationary iteration method under suitable constraint conditions, we derived the approximated solutions of μ and R on a single scattering model. During the iteration, the estimated value of μ can be rectified by introducing the large variations of R, whereas the small ones were automatically ignored. Based on the calculation of the structure information, the OCT intensity with attenuation compensation was deduced and compared with the original OCT profiles.

**Results:**

The preliminary validation was performed in the OCT A-line simulation and Monte Carlo modeling, and the subsequent experiment was conducted on multi-layer silicone-dye-${\mathrm{TiO}}_{2}$ phantoms and *ex vivo* cow eyes. Our method achieved robust and precise estimation of μ and R for both simulated and experimental data. Moreover, corresponding OCT images with attenuation compensation provided an improved resolution over the entire imaging range.

**Conclusions:**

Our proposed method was able to correct the estimation bias induced by the variations of R and provided accurate depth-resolved measurements of both μ and R simultaneously. The method does not require prior knowledge of the morphological information of tissue and represents more real-life tissues. Thus, it has the potential to help OCT imaging based disease diagnosis of complex and multi-layer biological tissue.

## 1 Introduction

Optical coherence tomography (OCT)^1^ can acquire high resolution cross-sectional images of biological samples by measuring their backscattered signals and has become a widely used tool for microstructure analysis,^2^ disease diagnosis, and biomedical imaging.^3^^,^^4^ However, OCT image contrast comes from the small range of in-sample refractive index variations, around 1.3 to 1.5,^5^ and this makes accurate real-time assessment of biological tissue challenging. To obtain accurate tissue anatomical structure information, there has been a growing interest in OCT attenuation coefficient imaging,^5^^,^^6^ which measures the light decay associated with the absorption and scattering. Following Lambert–Beer’s law, the irradiance L exhibits an exponential decay along the penetrating depth z, which can be written as

$$L(z)=L(0)e^{-2\int_{0}^{z}\mu (u)du},$$ (1)

where L(0) is the initial irradiance value, μ is the attenuation coefficient. With a low L(0), a large z or μ, or a combination of these factors, the decayed irradiance L(z) could be low, significantly limiting the visibility in deeper layers.^7^^,^^8^ Many OCT attenuation coefficient models have been developed to correct this attenuation loss and increase potential contrast for different tissue characterization, such as assessing the skin wound healing,^9^ atherosclerotic plaques,^10^^,^^11^ and filtering blebs after trabeculectomy.^12^ To quantify the attenuation of tissue, one straightforward approach is to assume a homogeneous sample and fit the OCT A-scan signals with exponential decay functions.^7^^,^^13^ Due to the noise reduction and pre-averaging step, the attenuation coefficient is not depth-resolved, resulting in a loss of details of the inner structures of samples.

Inspired by ultrasonic imaging techniques,^14^ a depth-dependent model was proposed to measure local attenuation properties.^15^^,^^16^ It bypasses the fitting step and noticeably outperforms in turbid samples with strong axial variations in light scattering. Later, several methods have been designed to improve upon this method by point spread function (PSF)^17^^,^^18^ as a practical light source and taking the noise floor into account.^19^ Another type of improved algorithms have been developed to mitigate the distal end errors,^20^^,^^21^ which is caused by the Vermeer’s model hypothesis (L(∞)=0). However, all these computational approaches assumed a constant backscattering of the attenuated light, denoted as the backscattered fraction R(z). In theory, R(z) depends strongly on the particle size, relative refractive index,^22^ and the numerical aperture (NA) of the OCT system.^23^ Previous studies proved that there is a significant difference in the backscattered fraction measured from intravascular OCT images.^24^ Therefore, the assumption of fixed R(z) can result in considerable under- or overestimation of tissue attenuation. More recently, Cannon et al.^22^ proposed an improved model that can measure the depth-resolved attenuation coefficients and the layer-resolved backscattering fractions simultaneously, and thus able to reduce measurement errors for heterogeneous samples. However, this method requires pre-segmentation of each layer, and an inaccurate segmentation could seriously affect the subsequent attenuation estimation. Furthermore, it is still challenging to precisely measure μ(z) for a complex tissue containing fuzzy boundaries and noise.

In this study, we make a systematical analysis of over- and underestimation of attenuation coefficients μ(z) due to the assumption of a constant backscattering fraction and present an alternative and practical depth-dependent attenuation characterization method without requiring a pre-segmentation step. As shown in Eq. (1), the decay rate of irradiance L(z) relies on μ(z), whereas the acquired OCT intensity is determined by the product of all three variables, μ(z), R(z), and L(z). To decouple their contributions, an underdetermined equation set with initial conditions was deduced. Approximated μ and R were then solved by iterations and updated simultaneously. Several constraint conditions were introduced to control the iteration process in practice. The stability and convergency of the equation set is discussed in the later section. Based on the estimated optical properties of samples, a model for correcting the light attenuation loss is also demonstrated. Our method is first verified using numerically simulated OCT A-scan signals and Monte Carlo simulated numerical phantom models. Further validations are performed using multi-layer silicone-dye-${\mathrm{TiO}}_{2}$ phantoms and bovine retina imaged with our in-house built SS-OCT (sweep-source OCT). Compared with raw OCT images of the retinal tissue, our attenuation-corrected OCT profiles are more accurate assessing the retinal microvasculature. We believe that such an accurate tissue characterization of μ and R and the attenuation compensation model can be a highly effective diagnostic tool for analyzing complex and heterogeneous biological samples.

## 2 Method

### 2.1 Theory

#### 2.1.1 Iteration-based computation of μ and R

As shown in Eq. (1), the irradiance value L ($W/{\mathrm{mm}}^{2}$) decreases when the light propagates through a medium with the loss. In our SS-OCT system, the acquired digital signal intensity I(z) detected at the depth z is a portion of the irradiance value, given as^15^

$$I(z)=-\beta R(z)\frac{dL(z)}{dz}=2\beta R(z)\mu (z)L(z),$$ (2)

where β is a converting factor related to the detection quantum efficiency and digitization process.^23^ Note that the axial PSF calibration, sensitivity roll-off, and noise floor related to I(z) in our SS-OCT system are discussed later in Sec. 2.3. The factor 2 is due to the round trip between the detector and the sample. According to the scattering theory, the attenuation coefficient μ is determined by the scattering coefficient $\mu_{s}$ and absorption coefficient $\mu_{a}$. $\mu_{a}$ is negligible compared to $\mu_{s}$ in the near-infrared region.^25^ $\mu_{s}$ depends on the geometric size of the scattering particle, their volume density, the incident wavelength, and refractive indices.^26^ On the contrary, R(z) is not affected by the volume density and is only determined by the other factors above. For an ideal sphere case, it can be written as

$$R=\frac{2\pi \int_{\arcsin(NA)}^{\pi }\gamma (\theta )\sin\;\theta d\theta }{2\pi \int_{0}^{\pi }\gamma (\theta )\sin\;\theta d\theta },$$ (3)

where θ is the angle between the incoming wave and backscattered wave (θ>90  deg), and γ(θ) is the volume scattering function, indicating the differential scattering cross section of particles per unit volume.^23^^,^^27^ The backscattering fraction R(z) is the ratio of the integral of γ(θ) over the acceptance angles determined by NA to the integral of γ(θ) over full solid angle. Another relevant parameter is the scattering anisotropy, defined as the average cosine of the scattering angle

$$g=\frac{2\pi \int_{0}^{\pi }\gamma (\theta )\sin\;\theta \;\cos\;\theta d\theta }{2\pi \int_{0}^{\pi }\gamma (\theta )\sin\;\theta d\theta }.$$ (4)

In the following section, we do not discuss the calculation of g. It is only used for analyzing the scattering behaviors of samples and building a numerical phantom using the Monte Carlo simulation.

Here, we assume the sample surface is at depth 0 and the light decays to zero at infinity. The penetration depth is also unlimited. Similar to Eq. (2), the initial value of the signal intensity I(z) can be written as

I(0)=2βR(0)μ(0)L(0). (5)

Next, we continue the work by Vermeer’s method^15^ and introduce an integral term of I(z) from Eq. (2):

$$\int_{z}^{\infty }I(u)du=-\beta \int_{z}^{\infty }R(u)\frac{dL(u)}{du}du=\beta R(z)L(z)+\beta \int_{z}^{\infty }\frac{dR(u)}{du}L(u)du.$$ (6)

Equation (6) is deduced by the integration by parts. Following Ref. 15, we divide Eq. (2) by Eq. (6) and substitute L(z) by Eq. (1), we obtain

$$\frac{I(z)}{2\int_{z}^{\infty }I(u)du}=\frac{R(z)\mu (z)L(z)}{R(z)L(z)+\int_{z}^{\infty }\frac{dR(u)}{du}L(u)du}.$$ (7)

To better separate μ(z) from other variables, we move them to left-hand side (LHS), shown as

$$\;\mu (z)=\frac{I(z)}{2\int_{z}^{\infty }I(u)du}\times (1+\frac{\int_{z}^{\infty }\frac{dR(u)}{du}L(u)du}{R(z)L(z)}),$$ (8)

$$=\;\frac{I(z)}{2\int_{z}^{\infty }I(u)du}\times (1+\frac{\int_{z}^{\infty }\frac{dR(u)}{du}e^{-2\int_{0}^{u}\mu (v)dv}du}{R(z)e^{-2\int_{0}^{z}\mu (v)dv}}).$$ (9)

If the backscattering fraction is assumed constant, the derivatives of R(z) will be zero and Eq. (9) will degenerate to the basic attenuation coefficient estimation model,^15^ given as

$$\mu (z)=\frac{I(z)}{2\int_{z}^{\infty }I(u)du}.$$ (10)

For multi-layer samples with varying backscattering fractions, using the simplified model in Eq. (10) can lead to both under- and overestimation of μ(z) over the entire imaging range. Both R(z), μ(z), and the derivative of R(z) can affect this type of measurement errors. To calculate the backscattering fraction R(z), we unitize the model introduced by Liu et al.^24^ It subdivides a heterogeneous sample into portions small enough to be homogeneous at any depth, e.g., $z\in (z_{1},z_{2})$, where $\mu (z)=\mu (z_{1})$ nd $R(z)=R(z_{1})$. Under this condition, Eq. (2) can be modified as

$$I(z)\approx 2\beta R(z_{1})\mu (z_{1})L(z_{1})e^{-2(z-z_{1})\mu (z_{1})},$$ (11)

where I(z) at depth $z\in (z_{1},z_{2})$. $e^{-2(z-z_{1})\mu (z_{1})}$ is treated as 1 since its thickness is small. We take the logarithm of Eq. (11) and plug Eq. (1) into it, and obtain

$$\mathrm{In}\;2\beta L_{0}\cdot R(z_{1})\approx \mathrm{In}\;\frac{I(z)}{\mu (z_{1})}+2\int_{0}^{z_{1}}\mu (u)du,$$ (12)

where the logarithm of R(z) has a linear relationship both with the integral of μ(z) and the logarithm of I(z) divided by μ(z). This assumption simplifies calculations and ensures an accurate estimation of R(z) in a discrete form.

Equations (9) and (12) form a nonlinear integral equation set, which describes a strong coupling between R(z) and μ(z) and their contributions to the acquired OCT signal intensity, given as

$$\left\{ \begin{array}{l} \;\mu (z)=\;\frac{I(z)}{2\int_{z}^{\infty }I(u)du}\times (1+\frac{\int_{z}^{\infty }\frac{dR(u)}{du}e^{-2\int_{0}^{u}\mu (v)dv}du}{R(z)e^{-2\int_{0}^{z}\mu (v)dv}})\\ \mathrm{In}\;2\beta L_{0}\cdot R(z_{1})\approx \mathrm{In}\;\frac{I(z)}{\mu (z_{1})}+2\int_{0}^{z_{1}}\mu (u)du \end{array}.\right.$$ (13)

They are transcendental equations including exponents and logarithms and thus there are no explicit general solutions. Moreover, the whole equation set is underdetermined since they are both derived from Eq. (2). To address this, we adopted an extended form of a stationary iterative method to obtain an approximated solution to this nonlinear system, where only a single unknown variable μ(z) is on the LHS in Eq. (13). It is defined as Richardson iteration,^28^

$$\mu_{k+1}(z)=M\mu_{k}(z)+c,$$ (14)

where k is the number of iterations, M is the iteration matrix, and c is a constant vector. Its key idea is the successive approximation from $\mu_{k}$ to $\mu_{k+1}$, where $\mu_{k+1}$ only depends on $\mu_{k}$ but not the previous values $\mu_{0,1,\ldots ,k-1}$. The initial values $\mu_{0}(z)$ and $R_{0}(z)$ are calculated by Eqs. (10) and (12), respectively. The digitized irradiance value βL(0) is acquired at the beginning of experiments, detailed in Sec. 2.3. Owing to the strong coupling in Eq. (13), several constraint conditions are added to better approximate the ground truth $\left\{\mu_{g}(z),R_{g}(z)\right\}$. Without them, the variables $\left\{\mu_{k}(z),R_{k}(z)\right\}$ might remain unchanged after initialization, resulting in infinite but similar loops (detailed in Sec. 2.1.2). As stated in Eq. (13), the recursion from $\mu_{k}$ to $\mu_{k+1}$ is determined by the derivative of the backscattering fraction $\frac{dR(z)}{dz}$, and any level of changes in R(z) at depth z can influence the iteration procedure ahead of it. As a result, $R_{k}(z)$ is fuzzily processed and only large changes in $R_{k}(z)$ are retained. The rate of changes of $R_{k}$ was measured by $\frac{dR_{k}(z)}{dz}$. It is reasonable to ignore the small signal fluctuation, because it may come from measuring errors, such as the system speckle noise, whereas the large variation is caused by the sample structures. Through multiple tests, we confirmed that four to five times the average derivative $\frac{\overset{˜}{dR_{k}(z)}}{dz}$ was normally an appropriate threshold for all the following simulations and experiments to reduce the noise and preserve the signal edge. Each region with small variation of $R_{k}$ was substituted by its average value. Due to the strong coupling between $\mu_{k}$ and $R_{k}$, omitting this fuzzy processing step or using a very small threshold can result in duplicated outputs in each loop. On the contrary, if changes of $R_{k}$ are not considered, our estimation equation will degenerate to the basic attenuation estimation model in Eq. (10).

#### 2.1.2 Convergence and constraint conditions

The convergence of our equation set is determined by its iteration matrix. In a linear system, for all initial $\mu_{0}$, the convergent solution is proved to exist when the induced norm of iteration matrix M satisfies that ‖M‖<1.^28^ In the Supplemental Material, we demonstrated that the induced norm can be larger than 1 in certain cases. Therefore, there is no guarantee that a global convergent solution will be obtained. However, local optimal values can be approximated given that the initial value $\mu_{0}(z)$ by Vermeer’s model^15^ is usually close to ground truth value $\mu_{g}(z)$, which has the same order of magnitude. Here, we added practical constraints on the iteration process to prevent unnatural distortions of μ(z) and R(z), given as

R(z)∈(0,1), (15a)

$$R(z)\in (A\cdot \overset{˜}{R},B\cdot \overset{˜}{R}),\overset{˜}{R}=\;\frac{\int_{0}^{\infty }I(z)dz}{\beta L_{0}},$$ (15b)

$$\frac{\mu_{k}(z)}{\mu_{0}(z)}\in (C,D),$$ (15c)

k<E, (15d)

$$|\mu_{k+1}(z_{0})-\mu_{k}(z_{0})|>0.01\times \mu_{k}(z_{0}),\;z_{0}=\underset{z}{\max}\;|\mu_{k+1}(z)-\mu_{k}(z)|.$$ (15e)

{A,B,C,D,E} are the scaling factors that can be adjusted according to an actual situation. Equation (15a) is based on the range of R(z), which is a unitless ratio between 0 and 1. Equation (15b) describes the fluctuation of depth-dependent R(z) around its average value $\tilde{R}(z)$ computed by Eq. (2). Equation (15c) prevents overcompensation of the attenuation μ(z). As stated in Eqs. (15d) and (15e), the program will stop executing once it achieves a local convergence or the loop is completed after a predetermined iteration value.

#### 2.1.3 Attenuation compensation model

As shown in Eq. (2), the acquired signal intensity I(z) is badly affected by the decayed irradiance L(z), leading to the deeper region of thick specimens undetectable. On the contrary, the ideal signal intensity $I_{D}(z)$ without the influence of the attenuated irradiance of the layers above can be assumed as

$$I_{D}(z)=2R(z)\mu (z)\beta L(0),$$ (16)

where the initial irradiance L(0) takes place of the attenuated irradiance L(z). With more accurate μ(z) and R(z) obtained by our model, we could achieve a better intensity compensation $I_{D}(z)$ for the attenuation loss in OCT images. Previous studies^9^^,^^29^^,^^30^ took the attenuation map μ as a representation of the compensated intensity map $I_{D}$, where $I_{D}(z)$ is proportional to μ(z) with R(z) being a constant. This is not applicable to a multi-layer tissue with complex scattering behaviors. Our model works better on heterogeneous samples by taking the backscattering fraction into intensity compensation, which allows a more precise reconstruction of OCT signal intensity.

### 2.2 Manufacturing Process of Samples

To validate the performance of our method, two-layer silicone elastomer-based phantoms were fabricated using a well-known protocol.^31^ First, viscous suspensions including ${\mathrm{TiO}}_{2}$ particles, a black dye (Higgins^®^), and a base elastomer (Sylgard^®^ 284 Silicone Elastomer MicroLubrol) along with a curing agent were well blended. Different backscattering properties were obtained by changing the concentration of ${\mathrm{TiO}}_{2}$ particles and the dyes, which has a different dependency on the light scattering and absorption.^31^ The mass concentration of ${\mathrm{TiO}}_{2}$ and a black dye are set to 0.1 w%, 0.2 w%, 0.25 w%, 0.3 w%, and 0.5 w%, 0.75 w%, 1.5 w%, 2 w%, respectively. The thickness of each cured mixture was around 300 to 600  μm. After curing, they were imaged and stacked into different two-layer phantoms. The tissue study was conducted using *ex vivo* healthy bovine retinas, which were dissected to obtain an open-sky view for OCT imaging.

### 2.3 OCT System Setup and System Calibration

An in-house built SS-OCT system that operated at a 100 kHz sweep rate with a sweeping range of 100 nm and a center wavelength of 1060 nm was used for all experiments. The axial resolution was ∼6  μm in air and ∼4.5  μm in biological samples, whereas the lateral resolution was ∼12.9  μm. The NA of our OCT system is 0.05. Taking axial PSF and the sensitivity roll-off into account, the OCT signal intensity defined in Eq. (2) is modified as

$$I_{\text{raw}}(z)\propto \beta \cdot H(z-z_{f},z_{R})\cdot T(z)\cdot R(z)\mu (z)L(z),$$ (17)

where T(z) and $H(z-z_{f},z_{R})$ denote the sensitivity roll-off and the axial PSF, respectively. $z_{f}$ is the depth of focus related to the sample surface. Benefited from the long coherence length of our system, ∼12  mm, the effect of the sensitivity roll-off is negligible within the sample imaging depth of ∼3  mm. Moreover, $H(z-z_{f},z_{R})$ is expressed as^32^

$$H(z-z_{f},z_{R})={\left({\left(\frac{z-z_{f}}{z_{R}}\right)}^{2}+1\right)}^{-1}.$$ (18)

Here, $z_{R}$ denotes the Rayleigh length of the incident Gaussian beam. A highly reflective sample was imaged twice at different depths,^33^ whose signal intensities were denoted as image1 and image2. The difference of the surface depth in these two images was measured as Δz. According to Eq. (17), the normalized signal intensity $I_{\text{raw}}(z)/H(z-z_{f},z_{R})$ of image1 should be equal to the normalized signal intensity of image2 at the depth z+Δz. Therefore, the values of $z_{R}$ and $z_{f}$ can be estimated by exhaustive search to minimize the following equation:

{zR^,zf^}=arg minzR,zf ‖(Iraw(z)/H(z−zf,zR))image1−(Iraw(z+Δz)/H(z+Δz−zf,zR))image2‖1. (19)

The $L_{1}$ norm of all 1024 A-scans in the B-scan was computed to reduce errors and exclude outliers. The background noise is removed by subtracting the background signals obtained without the sample. The term βL(0) is acquired by the global integration of OCT signals (unit is A.U.) from a strong reflector, calculated as 20,068 A.U. × mm.

### 2.4 Signal Processing

**Fig. 1:**
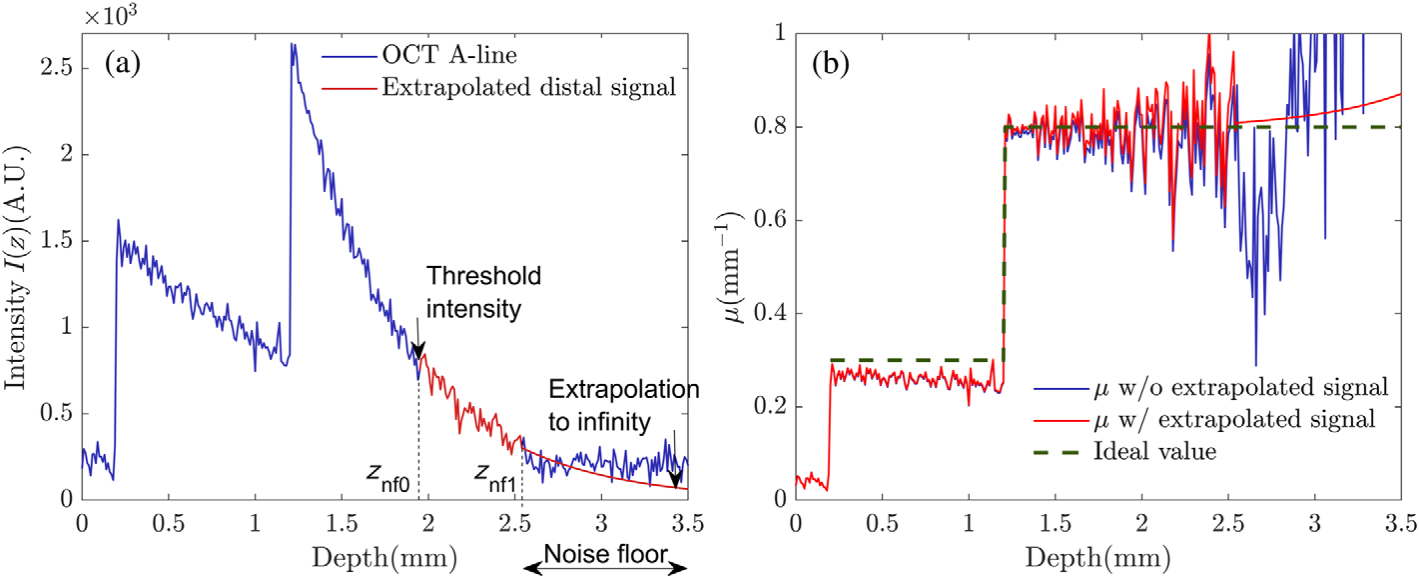
Example of locating and removing the noise floor by signal fitting extrapolation: (a) representative OCT signal intensity and (b) measured attenuation coefficient μ with and without extrapolation. The OCT signal intensity in red is extrapolated to infinity.

Our approach is based on the iterative calculation and all the real-time OCT data were saved for offline processing using Matlab. The first step is to remove the axial PSF $H(z-z_{f})$ from the acquired signal intensity $I_{\text{raw}}(z)$. The averaging is used to remove the speckle noise and increase SNR with a temporal window size of 5 B-mode images. Then, the signal intensity of the noise floor is identified and replaced by a fitting signal, for example, as shown in Fig. 1(a). The intensity of the noise floor at depth $z_{nf1}$ is defined as 50 dB. The average attenuation coefficient $\tilde{\mu }$ near the distal end is obtained by fitting the signal intensity at the depth $z\in (z_{nf0},z_{nf1})$, which is given as

$$I(z)\propto \overset{˜}{\mu }e^{-2\overset{˜}{\mu }z},\;z\in (z_{nf0},z_{nf1}),$$ (20)

where the intensity at the starting point $z_{nf0}$ is defined as 58 dB. The intensity of the noise floor I(z) deeper than $z=z_{nf1}$ is extrapolated to infinity from Eq. (20). The curve fitting and extrapolation can reduce the effect of the noise floor; otherwise it can cause the underestimation of μ^19^ when using Eq. (10), as shown in blue in Fig. 1(b). Moreover, the basic assumption of Eq. (10) is satisfied since the predictive signal intensity travels deep enough that the irradiance light decays away completely at that penetration depth.^15^ Because of neglecting the varying R, the measurement of $\mu_{0}$ based on Eq. (10) deviates from the ideal value. Next, initial values $\left\{\mu_{0}(z),R_{0}(z)\right\}$ are computed using Eqs. (10) and (12), whereas the average backscattering fraction $\tilde{R}(z)$ of the sample is computed (detailed in Sec. 2.1.2). Once the program is executed, the stationary iteration method is utilized to resolve a transcendental equation set Eq. (13), by a successive approximation of $\left\{\mu_{k}(z),R_{k}(z)\right\}$. To decouple the equation set, the backscattering fraction $R_{k}(z)$ is blurred in each loop. Only larger changes of $R_{k}(z)$ at a specified threshold level are taken into account to rectify the measurement error of $\mu_{k}(z)$. The program stops automatically if one of the criteria is not met. Finally, the optimized tissue profiles $\left\{\mu_{k}(z),R_{k}(z)\right\}$ is used for calculating OCT images with attenuation compensation $I_{D}$ based on Eq. (16). The detailed sequence processing is shown in Algorithm 1.

**Algorithm 1:** Tissue characterization by stationary iterative method

**Input:** conventional tomogram reconstruction $I_{\text{raw}}(z)$, system parameters H(z), βL(0).	
**Output:** estimated attenuation coefficient $\mu_{k}(z)$, backscattering fraction $R_{k}(z)$ after iteration, compensated tomogram reconstruction $I_{D}(z)$.	
1: System calibration and averaging	$I(z)=\frac{1}{N}\overset{N}{\underset{i=1}{\sum }}{\left[I_{\text{raw}}(z)\cdot H(z{)}^{-1}\right]}_{j}$
2: Locate and remove the noise floor by signal fitting extrapolation	/*detailed in Eq. (20)*/
3: Compute initial values $\left\{\mu_{0}(z),R_{0}(z)\right\}$ and average backscattering term $\tilde{R}$	/*Calculation of $\mu_{0}(z)$ is based on Eq. (10)*/
	$R_{0}(z)\approx \exp(\mathrm{In}\;\frac{I(z)}{\mu (z_{1})}+2\int_{0}^{z_{1}}\mu (u)du)\cdot {\left(2\beta L_{0}\right)}^{-1};$
	/* Calculation of $\tilde{R}$ is based on Eq. (15b)*/
4: **while** constraints are true **do**	/*Detailed in Eq. (15)*/
5: $\left\{\mu_{k}(z),R_{k}(z)\}\to \{\mu_{k+1}(z),R_{k+1}(z)\right\}$	/*$R_{k}(z)$ is fuzzily processed. Detailed in Eq. (13)*/
6: **end**	
7: Compute attenuation compensated tomogram $I_{D}(z)$	/*Detailed in Eq. (16)*/

### 2.5 Numerical Simulation

#### 2.5.1 A-mode numerical imaging

A numerical simulation of the OCT depth profile was performed using a single scattering model by defining μ and R. The calculation was based on Eq. (2), with a constant value βL(0)=2.07E−2(A.U.×pixel). Each digital phantom contained four layers and their bottom layers were set to be infinitely long, where all the light decayed to zero. All phantoms were placed at a depth of 125  μm, with an axial resolution of 1  μm. The A-line simulation depicted an ideal situation that avoided the estimation bias of μ, which neglected many interference factors, including the multiple scattering effects, a limited imaging depth of the system, the speckle noise, and the noise floor. The ground truth values $\mu_{g}$ of the region $z\in (z_{1},z_{2})$ were computed by fitting I(z) with an exponential function in Eq. (20); the ground truth values $R_{g}$ was computed by Cannon’s layer-based model as

$$R_{g}(z)=\frac{2\int_{z_{1}}^{z_{2}}I(z)dz}{\beta L(z_{1})(1-e^{-2\mu_{g}(z_{2}-z_{1})})}.\;z\in (z_{1},z_{2}).$$ (21)

Ground truth values are also computed in the same way in the subsequent experiments.

#### 2.5.2 Monte Carlo simulation

The basic theory of Monte Carlo modeling for light transport (MCML) is well-described by Jacques and Wang,^34^ and it is a powerful tool for analyzing laser–matter interactions, especially for multi-layer materials. Here, we chose the Monte Carlo approach to simulate the light propagation through a complex layered retinal model and applied our method for tissue characterization. The retinal model has a complex layered structure,^35^ and it is a suitable model to evaluate and compare different attenuation characterization models. The overall geometrical retinal model is shown in Table 1. The 13-layer geometry was based on the common four-layer retinal model containing retina, retinal pigment epithelium (RPE), choroid with 70% blood, and sclera.^36^^,^^37^ To subdivide the neural retina into 10 layers, 20 OCT images from five *ex-vivo* bovine eyes were collected and segmented manually. To preserve edges and reduce speckle noise, each image was then divided into 10 regions of interest (RoIs), with each RoI containing 100 A-scans. These A-scans were then averaged individually. In our 13-layer model, the thickness of each sublayer was determined according to the layer segmentation result and the common four-layer model.^36^^,^^37^ The absorption coefficient $\mu_{a}$, scattering coefficient $\mu_{s}$, and anisotropy g of each sublayer were determined by using an exhaustive search within plus or minus ∼50% average values of the neural retina from the original four-layer model^36^^,^^37^ to minimize the mean squared error between simulated A-lines and the average A-lines of each RoI. The simulated result was on a logarithmic scale with gamma correction. The simulation was conducted in the same open-sky view in order it to be consistent with our bovine retinal study, in which the eye was dissected and the vitreous was removed. The MCML algorithm was modified from Wang’s MCML programs.^34^ The weight of photon packages w is initialized as 1 and it decreases at each interaction step, due to effects such as photon absorption and scattering. $w_{r}$ is the total weight of phantoms that reflect from a reference arm, and $w_{s}$ is the total weight of phantoms that backscatter from the sample and fit the detecting condition (i.e., NA, detector position, and size). The detected OCT signal intensity I(z) is convolved by the following equation:^37^

$$I(z)=I_{0}\underset{i}{\sum }(\sqrt{w_{r}w_{s}(\Delta z_{i})}\;\cos(k(z-\Delta z_{i}))\exp(-{\left(\frac{z-\Delta z_{i}}{l_{\mathrm{coh}}}\right)}^{2})),$$ (22)

where $I_{0}$ is a constant value determined by the light source, $w_{r}$ and $w_{s}$ are the total weights of photons with optical path difference $\Delta z_{i}$, and $l_{\mathrm{coh}}$ is the coherence length of the light source. The related coefficients NA of 0.05, the coherence length of 5  μm, and the transversal scanning step of 10  μm were used. Four hundred consecutive A-lines were simulated with 5,000,000 weighted photons. No sensitivity roll-off in depth and axial PSF were considered here.

**Table 1:** Retina’s layers, thickness, and optical properties.

Layer	d (μm)	n	$\mu_{a}$ (1/cm)	$\mu_{s}$ (1/cm)	g
ILM	6	1.47	0.37	120	0.97
Retinal nerve fiber layer	5	1.47	0.37	120	0.97
GCL	19	1.47	0.40	114	0.97
IPL	27	1.47	0.37	134	0.98
Inner nuclear layer	20	1.47	0.34	130	0.97
OPL	20	1.47	0.38	166	0.98
ONL	60	1.47	0.31	110	0.98
ELM	4	1.47	0.38	136	0.97
Inner photoreceptor layer	12	1.47	0.29	134	0.97
Outer photoreceptor layer	27	1.47	0.9	357	0.93
RPE	10	1.47	80	1700	0.84
Choroid (70% blood)	250	1.47	0.75	500	0.94
Sclera	700	1.47	0.1	420	0.90

## 3 Result

### 3.1 Numerical Simulation

#### 3.1.1 Numerical simulation: A-mode

**Fig. 2:**
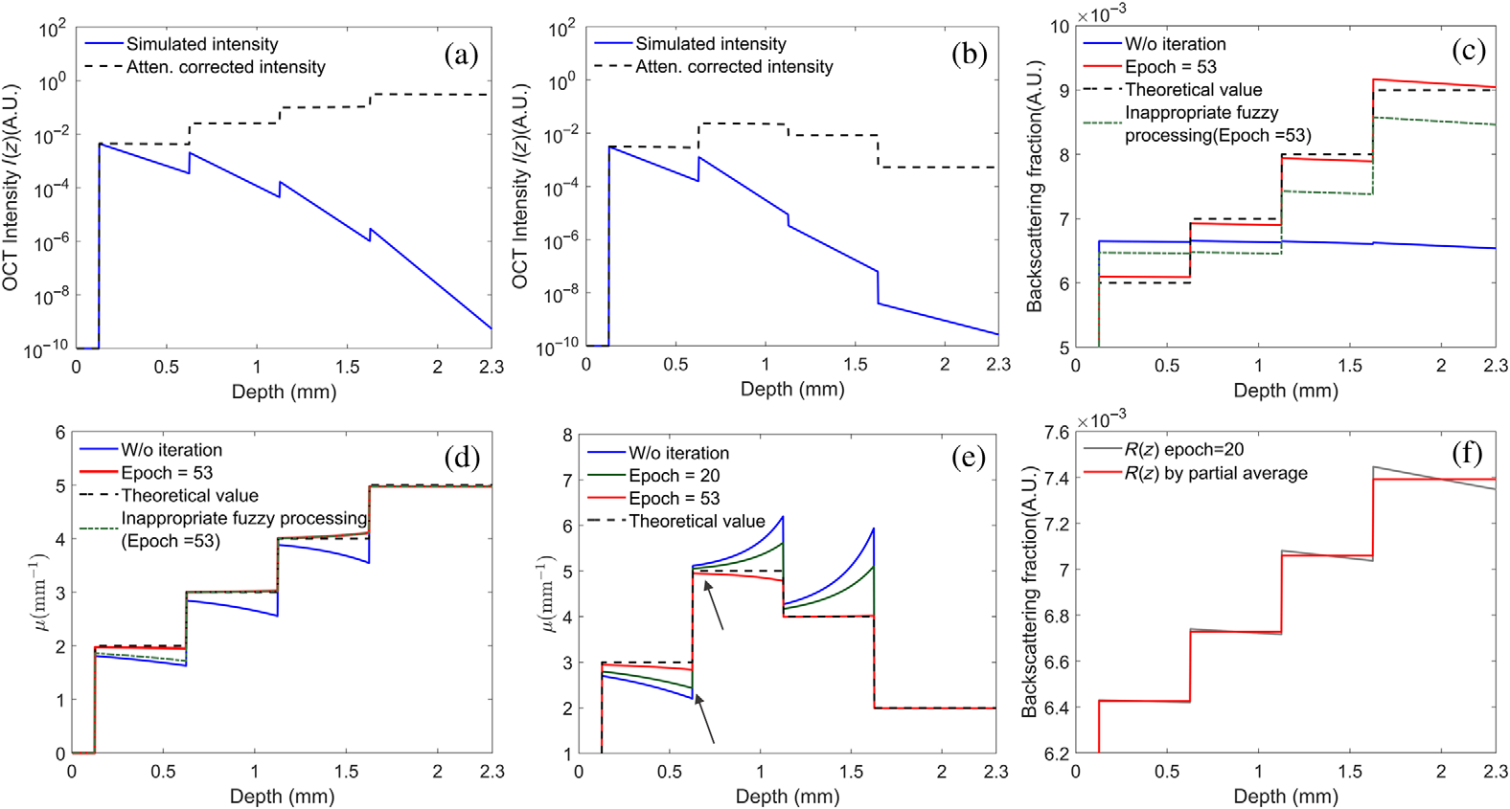
Numerical simulation result of (a), (b) OCT signal intensity of (a) first phantom and (b) second phantom before and after signal compensation on a logarithmic scale; (c) measured backscattering fraction R of the first phantom during iteration; (d), (e) measured attenuation coefficient μ of (d) first phantom and (e) second phantom with and without iteration; (f) example of the partial average algorithm when calculating backscattering fraction R of the first phantom. The ideal backscattering fraction R of the second phantom is 0.005, 0.007, 0.006, and 0.004, respectively. Other theoretical values are represented by dashed lines.

To initially test the feasibility of our method, two heterogeneous digital phantoms, each containing four layers, were simulated, with a thickness of 0.5 mm for the top three layers. The attenuation coefficient μ and backscattering fraction R of each layer were set to be distinct. No additional constraints between μ and R were established. As shown in Figs. 2(a) and 2(b), the logarithmic OCT A-line intensity I(z) (square of magnitude) decreases linearly, which agrees with Eqs. (1) and (2). The simulated backscattering fraction R of the first phantom is plotted in Fig. 2(c). The attenuation profiles measured by the conventional model using Eq. (10),^15^ and our algorithm is plotted in Figs. 2(d) and 2(e). Confounded by the layer-dependent R of samples, a large estimation bias can be seen using the conventional method without iteration ($\mu_{0}$). The amount of measurement bias varies across the depth, and it is more pronounced near the boundary of each layer, which is marked in gray. Note that this type of measuring error reduces to zero in the bottom layer, which indicates that the error at a certain depth is only related to the derivative of R below that point, as we previously analyzed in Eq. (9). On the contrary, we obtained a more accurate estimation result if we utilize the stationary iteration method for $\mu_{0}$ and substitute I(z)/R(z) for I(z). As shown in Fig. 2(c), the initial value $R_{0}$ appears to be constant within the whole penetrating depth, $R\approx 6.7\times 10^{-3}$, and deviates from the true value as shown by the dashed line. When the iteration starts, both the updated coefficients $\mu_{k}$ and $R_{k}$ closely match their theoretical values, shown in Figs. 2(c)–2(e). As shown in Figs. 2(c) and 2(d), if the threshold of $\frac{dR_{k}(z)}{dz}$ is not appropriately selected, for example, when it falls within the range of $80\overset{˜}{\frac{dR_{k}(z)}{dz}}$ and $\tilde{85\frac{dR_{k}(z)}{dz}}$, the inter-layer variations at ∼600  μm will be neglected by our algorithm. Moreover, it leads to a significant underestimation of the attenuation coefficient μ within the range of 0 to 600  μm and R across the entire imaging depth. However, even under ideal conditions without Gaussian noise, our method cannot achieve zero estimation errors between $\left\{\mu_{k},R_{k}\right\}$ and true values $\left\{\mu_{g},R_{g}\right\}$ as shown in Figs. 2(c) and 2(e). These discrepancies stem from the absence of global convergence of our algorithm (see the Supplemental Material for details). Figure 2(f) shows the local averaging step of $R_{k}$. It keeps larger variations near boundaries while ignoring small ones caused by the local structures of tissues and noise, which improves the stability of our algorithm.

After the optical properties were corrected, we applied the attenuation compensation method given by Eq. (16) to correct the OCT signal intensity. As shown in Figs. 2(a) and 2(b), the intensity distribution with attenuation compensation is consistent with the distribution of the samples’ optical properties with distinct μ and R. Compared to the initial OCT A-line intensity, it provides more accurate morphological information over the entire imaging depth without the attenuation loss.

#### 3.1.2 Monte Carlo simulation: B-mode

**Fig. 3:**
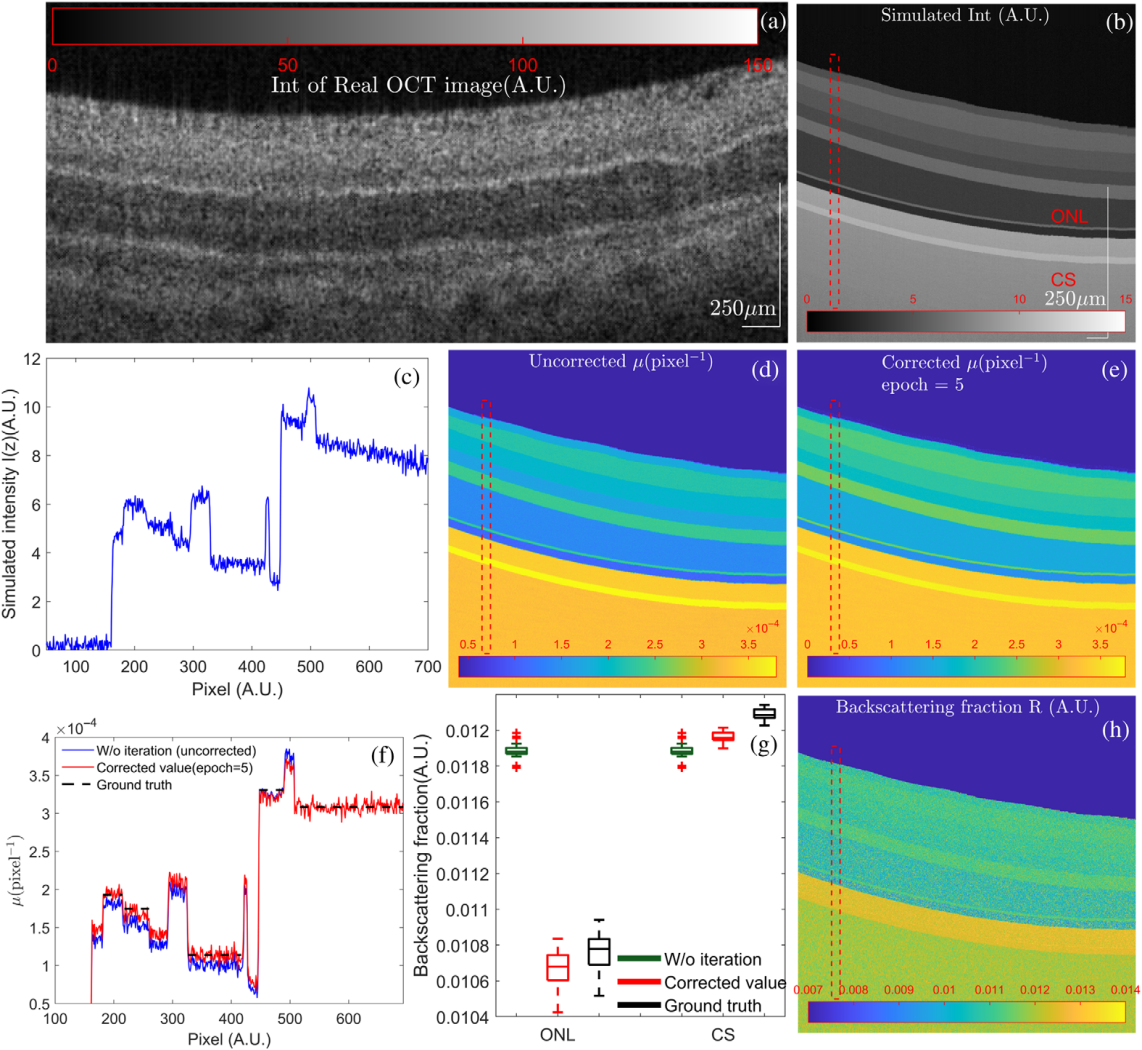
(a) The experimentally obtained real OCT image of the bovine retinal tissue for comparison. (b) The simulated OCT image of the bovine retinal tissue obtained using Monte Carlo modeling. The image size is set to 800×400  pixels with resolutions of 0.7  μm per pixel axially and 10  μm per pixel laterally. (c) A representative OCT A-line signal within RoI indicated as a red dashed box in (b). (d)–(f) Measured attenuation coefficient μ and representative A-line μ of the red dashed RoI with and without iterations. (g)–(h) The comparison between measured backscattering fraction R of the red dashed RoI and the measured backscattering map obtained using our iterative method.

Based on the Monte Carlo modeling mentioned above, we recorded the photon trajectory and the corresponding OCT intensity of the retinal tissue model. The simulated results have a distinct layered structure that matches real samples as shown in Figs. 3(a) and 3(b). Compared to frequently used four-layer retinal model, our modified version has a complex layered structure and matches our *ex-vivo* samples better, as shown in Figs. 3(a) and 3(b). The regions above the internal limiting membrane (ILM) were air both in Figs. 3(a) and 3(b). A representative OCT A-scan image from dashed red RoI in Fig. 3(b) is shown as a blue line [Fig. 3(c)]. There is no significant exponential decrease within each layer because of its small thickness, absorption, and scattering coefficients, which makes it difficult to compute the ground truth $\left\{\mu_{g},R_{g}\right\}$ by a fitting curve. Therefore, we only fit the intensity distribution I(z) and computed the ground truth $\mu_{g}$ of five thicker layers, i.e., the ganglion cell layer (GCL), the inner plexiform layer (IPL), the outer nuclear layer (ONL), the photoreceptor outer segment layer, and the choroid. Due to a lower NA (NA = 0.05) and larger scattering anisotropy g, the signal intensity decays under the impact of multiple scatter light, leading to a smaller $\mu_{g}$, namely $\mu_{g}<\mu_{a}+\mu_{s}$.^23^

Figures 3(d)–3(h) demonstrate the backscattering fraction and attenuation maps and corresponding comparisons with theoretical values. To have a better comparison, we applied gamma correction^38^ (γ=0.75) to enhance the image contrast in attenuation map [Figs. 3(d) and 3(e)]. Governed by the scattering coefficients $\mu_{s}$ and anisotropy g, the ideal backscattering fraction $R_{g}$ first rises and then declines. It leads to various degrees of estimation biases of μ as shown in Fig. 2(f). Simulation results proved that our iteration method could calculate the samples’ depth-dependent backscattering fraction R and leverage it to rectify the over- or under-estimation of attenuation. We also found that iterations of our model stop with significantly fewer epochs when compared with the refractive-index-matched single-scattering A-line simulation in Fig. 2. This situation is consistent with our subsequent experimental results. A possible reason is that the multiple scattering, the axial PSF, and the scattering anisotropy^39^ work together to affect the OCT signal distribution, leading to a larger interlayer change of R. The adaptive iterative feedback of our algorithm could monitor the iteration procedure and stop it in time, which mitigates the effect of over-compensation. However, there still exists a slight error between $\left\{\mu_{k}(z),R_{k}(z)\right\}$ and the true values $\left\{\mu_{g},R_{g}\right\}$ in Figs. 3(f) and 3(g). They are caused by a lack of a global convergence of our algorithm (see the Supplemental Material for details). Suitable constraint conditions can improve the accuracy of our iterated approximating method.

### 3.2 Phantom Experiment

**Fig. 4:**
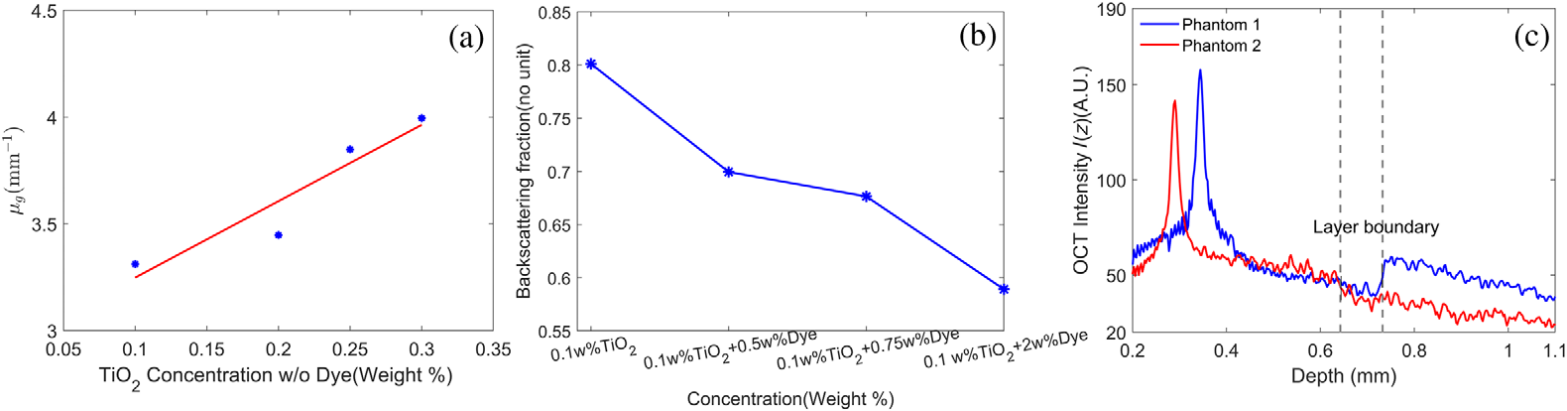
(a) Measured average attenuation coefficient μ that changes with the particle concentration by fitting an exponential curve. (b) Measured average backscattering fraction R that changes with the particle concentration using Eq. (21). The signal trend is marked by a solid line. (c) Representative OCT A-line signal intensity of the finalized home-made two-layer phantoms.

Eight distinct single-layer phantoms were constructed and imaged to evaluate our method. Note the specular reflection signals were removed. The calculations of the ideal values of $\mu_{g}$ and $R_{g}$ were discussed in Sec. 2.5.1, as shown in Figs. 4(a) and 4(b). The experiment results agree with the theoretical analysis that $\mu_{g}$ has a linear dependence on the scatterer concentration without the influence of the absorber (Pearson correlation coefficient ρ=0.948).^15^^,^^31^ More importantly, it demonstrates that the ratio of the absorber concentration to the scatter concentration has an adverse effect on the backscattering faction $R_{g}(\rho =0.957)$. To validate our method, these single-layer phantoms were stack together; examples of the A-line signal intensity is shown in Fig. 4(c). The signal intensity is rescaled between 0 and 255. The first layered phantom contained 0.1 w%, 0.3 w% of ${\mathrm{TiO}}_{2}$, 0.5 w%, 0 w% of the black dye, whereas the second layered phantom contained 0.2 w%, 0.1 w% of ${\mathrm{TiO}}_{2}$, 0 w%, 1.5 w% of the black dye, from the top to bottom.

**Fig. 5:**
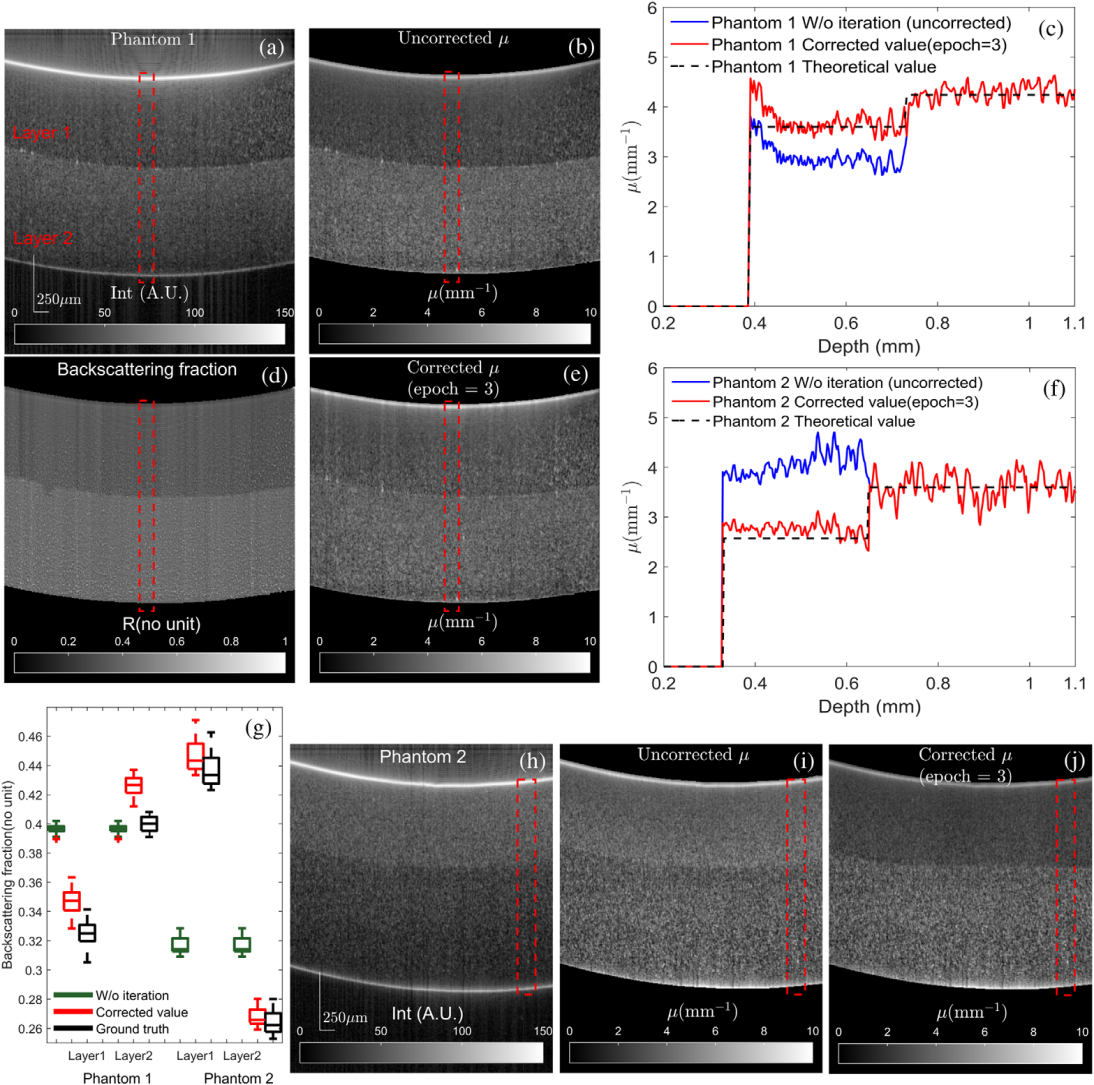
Phantom experiment results. (a) and (h) OCT signal intensity of the first and second phantoms. (b) and (i) Measured attenuation coefficient μ without iteration. (c) and (f) The comparison between calculated μ using different methods. (d) Measured backscattering fraction R of the first phantom using our method. (e) and (j) Measured attenuation coefficient μ with iteration. (g) The comparison between calculated R and ideal R in the dashed red RoI of (a) and (h).

Figures 5(a), 5(b), and 5(e) show OCT intensity and attenuation maps for the first phantom. A representative A-line shown by the red region is plotted in Fig. 5(c). Due to the increasing backscattering ratio $R_{g}$ [Fig. 5(g)], the attenuation profile $\mu_{0}$ in the top layer is underestimated severely. Simultaneously, we could notice a significant over-estimation bias in the same layer of the second phantom, making $\mu_{0}$ almost homogeneous [Fig. 5(i)]. On the contrary, the attenuation profiles $\mu_{k}$ using our method accurately matches the ground truth $\mu_{g}$ throughout the whole imaging depth as shown in Figs. 5(f) and 5(j). Our method is more robust against the backscattering variation and contains less estimation bias. Compared with other recent methods, it does not need a preliminary inter-layer segmentation, which is often difficult due to the fuzzy boundaries of OCT intensity profiles and the noise induced by the abnormal distributions of scatterers. Furthermore, it measures depth-dependent backscattering profiles as shown in Fig. 5(d). The map of $R_{k}$ shows significant inter-layer variations and very little intra-layer variations. The loss of the intra-layer resolution is caused by the averaging of $R_{k}$, where the local changes of $R_{k}$ are neglected during the iteration [Fig. 2(f)]. Overall, the respective mean backscattering profiles $R_{k}$ of all A-scans agree well with the ideal value $R_{g}$, as shown in Fig. 5(g). To mitigate the influence of the noise floor, the OCT intensity I(z) was extrapolated to infinity as explained earlier. Therefore, the measured $\mu_{k}$ and I(z) might not match. To avoid this scenario, $\mu_{k}$ below sample bottoms was set to zero.

### 3.3 Bovine Retinal Experiment

**Fig. 6:**
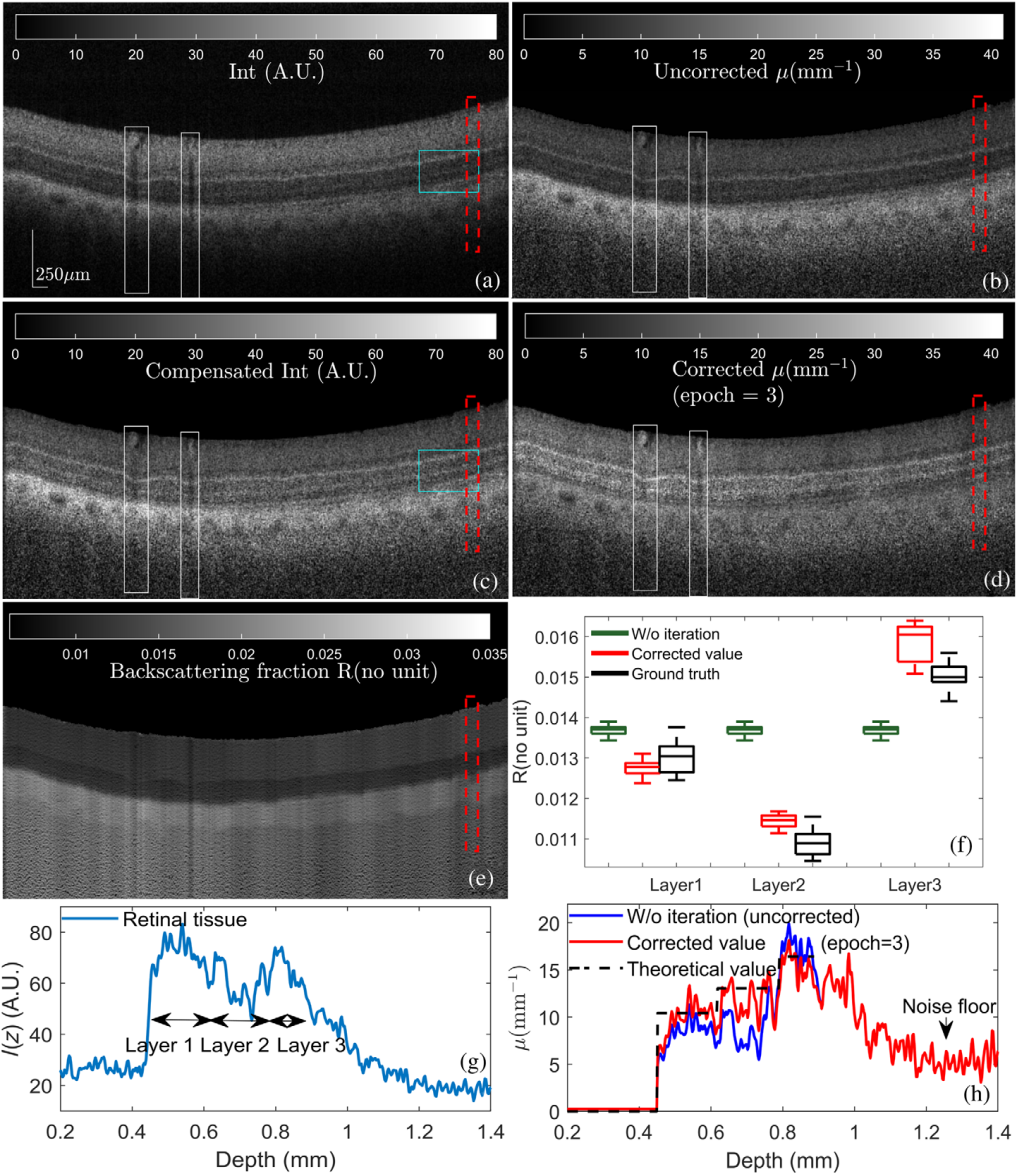
Experiment result of bovine retinal tissue. (a) and (c) OCT signal intensity of bovine retinal tissue (a) before and (c) after our intensity compensation method. (b) and (d) Measured attenuation coefficient μ (b) without and (d) with iteration. (e) Measured backscattering fraction R using our method. (f) The comparison between calculated R and ideal R of red RoI in (e). (g) Representative OCT A-line signal intensity of red RoI in (a). (h) The comparison between calculated μ using different methods of red RoI in (d).

To further test our method, an *ex vivo* bovine retinal tissue was imaged by our OCT system, as shown in Fig. 6(a). Figures 6(b), 6(d), and 6(e) show the corresponding attenuation and the backscattering fraction maps. Representative average A-lines of I, μ, and R of the red RoI are shown in Figs. 6(f)–6(h). Certain retinal layers can be quite thin, 17 to 30  μm^40^ thick with various types of cells, making it hard to precisely annotate the boundaries. The OCT signal intensity was divided into three parts and the ground truth values of μ in each part were computed by Eq. (20), respectively. The first layer consists of ILM through to outer plexiform layer (OPL), the second layer consists of ONL through to ELM, and the third layer is the outer retinal layer. No layer segmentation was conducted in our iterative method. As shown in Figs. 6(b) and 6(h), using the conventional model leads to over- and under-estimation errors of $\mu_{0}$ when neglecting inter-layer variations of $R_{g}$. Moreover, the measured attenuation profile $\mu_{0}$ of the outer retinal layer is severely polluted due to the blood vessel, which casts shadows over the bottom layers, highlighted by the white RoIs. Our model could track the large variation of $R_{k}$ adaptively and automatically, as shown in Fig. 6(e), to rectify the value $\mu_{k}$ given by Eq. (13). The mean values of $R_{k}$ precisely matched the ground truth values, as shown in Fig. 6(f). Figure 6(c) shows the processed OCT image with attenuation compensation by leveraging both $\mu_{k}$ and $R_{k}$, given by Eq. (16). Due to the light attenuation, the reflected signal intensity from the external limiting membrane (ELM) and sclera in Fig. 6(a) is faint and easily overshadowed by the speckle noise. On the contrary, our intensity compensation method [Fig. 6(c)] enhances their visibility, especially in the vascular area of the underlying choroid tissue, highlighted by the blue RoIs. It also eliminated shadow artifacts, and no overamplified background noise is observed when using our algorithm.

## 4 Discussion

We proposed and presented a depth-resolved attenuation estimation algorithm that calculates the depth-dependent backscattering fraction profile for SS-OCT imaging. The proposed method does not assume the backscattering fraction R as a constant in modeling the attenuation profiles and eliminates the under- and over-estimation problems it causes. Our proposed method provides a more precise characterization of tissue without explicit interlayer segmentation. The method is based on an iterative model using the nonlinear relationship between the irradiance, OCT intensity, and unknown optical properties of tissue. To decouple equations, our approach neglects the small variations in the backscattering fraction and applies appropriate constraint conditions for the solution convergence. It does not assume μ or R to be constant at any depth nor requires a layer segmentation preprocessing step. Furthermore, we utilized the corrected values of μ and R to compensate for the OCT signal intensity loss caused by the decreased irradiance, thus reducing the stripe noise and improving the global visibility of the OCT image.

The attenuation coefficient μ and the backscattering fraction R are two important characteristics of samples that could be derived from OCT images. Governed by the different combinations of scatterers’ characteristics, such as the geometric size, the volume density, and the nucleus, they represent the tissue characteristics from different perspectives.^24^ On the other hand, they tend to be highly coupled to interacting light and highly correlated with the governing equations. Therefore, their analytical solutions cannot be acquired directly without knowledge of other characteristics. To address this issue, the recent studies often measured them individually while assuming the other variables to be constant.^15^^,^^22^ These methods typically rely on accurate layer segmentation, which may fail to perform the task when there are gradual changes in tissue structure or extremely thin tissue layers, e.g., the retinal tissue. Inspired by these issues, we blurred the backscattering term R and utilized it to rectify the estimation bias of μ by iterative procedures. The fuzzy process maintains the significant variation of R while replacing the minor ones with a local average. The related thresholds can be adjusted automatically during each iteration, which applies to different ranges of R. This process is similar to a layer segmentation, except that it is adaptive with dynamic changes and only determined by R. Therefore, it is more sensible than the conventional intensity-based segmentation methods that are easily disturbed by both μ and R. It is often hard to segment the OCT intensity profile successfully when the effects of μ and R make boundaries blurry [shown in red in Fig. 4(c)]. During the iteration, the attenuation profile over the whole depth can be corrected automatically without the layer-by-layer analysis. Moreover, we believe that our method can be used in the visible spectrum when light absorption cannot be neglected. In this scenario, attenuation μ is measured as $\mu =\mu_{a}+\mu_{s}$, and R is measured as a ratio of the backscattered light to the attenuated light.^15^ Although the light absorption can reduce the magnitude of R, we can still rectify the measurement error of μ by computing $\frac{dR}{dz}$. We acknowledge that our existing fuzzy processing of R needs to be fine-tuned for tissue with less distinguishable layers, to extract the structure information from the noise. The metric of variation of R also needs to be improved. If these prerequisites are met, the attenuation profile over the entire depth can be corrected automatically by the derivative of R in Eq. (13) without the layer-by-layer analysis.

**Fig. 7:**
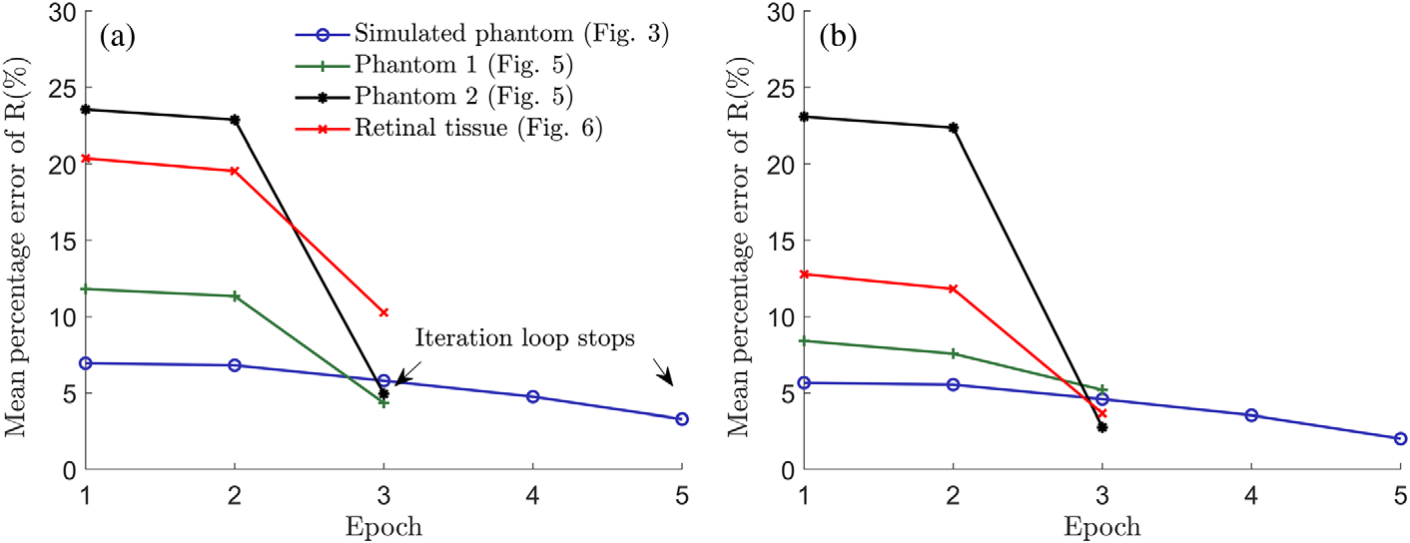
(a) The mean percentage errors between the estimated attenuation coefficients $\mu_{k}$ and true attenuation coefficients $\mu_{g}$ within all red dashed RoIs of phantoms and tissues in Figs. 3, 5, and 6. (b) The mean percentage errors between the estimated backscattering fractions $R_{k}$ and true backscattering fractions $R_{g}$ within all red dashed RoIs of phantoms and tissues in Figs. 3, 5, and 6.

It is crucial to design appropriate constraint conditions to control the loops when it has no global convergence (see the Supplemental Material). The empirical results indicate that two to four loops are typically enough to yield a reasonable solution for both representative phantoms and real tissues. In Fig. 7, we summarized the mean percentage errors between estimated values $\left\{\mu_{k},R_{k}\right\}$ and true values $\left\{\mu_{g},R_{g}\right\}$ obtained from all simulated and experimental results in Figs. 3, 5, and 6.

Based on Fig. 7, the first loop usually has relatively little impact on the accuracy. However, the second to fourth loops prove to be sufficient in reducing errors to below 10% for both tissues and phantoms used. Based on this information and the low complexity of each loop, our model has a strong potential for OCT-based real-time tissue assessment. One possible solution to enhance its performance is to convert the existing Matlab codes into a more efficient programming language. In addition, employing parallel processing with GPUs could further optimize the computation time, reducing it to hundreds of milliseconds per B-scan (1024 A-scans). Our proposed constraint conditions are also based on reasonable ranges of tissue’s optical properties, degree of compensation, and iterative times. For a wider application, we acknowledge that they might need further refinement to suit a particular tissue. The large drop in estimation errors near each interface can be another way to further refine constraint conditions, indicated by the black arrow in Fig. 2(e).

The presence of multiple scattering is also an important impact on the quantitative attenuation characterization, as we discussed in Figs. 3 and 6 of retina images. Under this circumstance, the high scattering anisotropy g and the small scattering angle jointly lead to a larger contribution of highly forward directed scattering behavior, with the rise of the backscattering fraction.^23^ It emphasizes the importance of our iterative computation model, which can reduce additive errors caused by the pixelwise variation of R. Our results suggest that multiple scattering may have more influence near each interface, and the effect on estimating μ could be reduced by the better control of the iterations. It is worth noting that backscattering fractions R calculated by our method can highlight the layer-to-layer boundaries, which is helpful in various clinical applications needing tissue layer segmentation. The further work might include analyzing the analytic solutions of optical properties under the multiple scattering modeling.

Our proposed method is evaluated through Monte Carlo simulation and OCT imaging. Both the iterative attenuation estimation model and the intensity-based compensation model demonstrate promising results over a wider depth, which can be advantageous for imaging heterogeneous tissues with a complex and subtle layered structure, such as intravascular tissue,^10^^,^^13^ retina,^41^ and brain.^42^

## 5 Conclusion

In this study, we presented a novel depth-resolved attenuation and backscattering analysis method that could remedy the estimation bias induced by depth-wise variations in backscattering fraction R. It also enables a depth-resolved calculation of the backscattering fraction, and an intensity-based compensation model is derived utilizing the corrected tissues’ optical properties. The methodology was validated through a simulated OCT A-line image, the Monte Carlo model, and experimental OCT imaging. The results demonstrated that this algorithm is capable of precisely estimating attenuation coefficients and the depth-dependent backscattering fractions in complex scattering samples, without any preliminary steps, such as layer segmentation. Therefore, it has great potential for the quantitative characterization of heterogeneous structures and for enlarging the related detectable regions of tissue for OCT imaging applications.

## Code, Data, and Materials Availability

The data that support the findings of this study are available from the corresponding author upon reasonable request.
