# Supplementary material for: Depth-dependent attenuation and backscattering characterization of optical coherence tomography by stationary iterative method
Source: J Biomed Opt. 2023 Aug 24;28(8):085002. doi: 10.1117/1.JBO.28.8.085002 (PMC10449262; doi:10.1117/1.JBO.28.8.085002)
Supplement: Supplementary file 1 [file JBO_028_085002_SD001.pdf]

Supplementary information

Analysis on the global convergence of our model by reductio

Our iterative attenuation estimation model is based on *Richardson iteration*[1], which is given as,

$$\mu_{k+1} = M\mu_k + c, \tag{14}$$

where k is the number of iterations, M is the iteration matrix, μ is the unsolved attenuation coefficient, and c is a constant vector. According to ref. [27], if the induced norm $||M|| < 1$, the convergent solution of Eq. 14 always exists for all initial μ_0, which is $\mu = (I - M)^{-1}c$. To discuss the convergence of our method, we need to rewrite our iterative equation set Eq. 13 in the form of Eq. 14. In order to simplify calculating, we transform Eq. 13 into a discrete form. The discrete attenuation coefficient $\mu(n)$ has a pixel size of Δ, and n is a natural number. The integral of $\mu(z)$ $\int_0^z \mu(v)dv$, could be expressed as $\sum_{p=0}^{n} \mu(p)\Delta\,, (n = z/\Delta)$. Similarly, Eq. 9 can be expressed as,

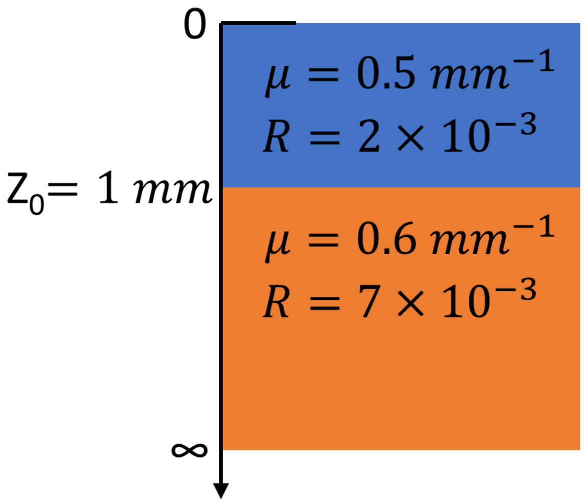

Figure S1. Thickness and optical properties of the sample phantom.

$$\mu(n)_{k+1} \approx \mu_0(n) \times \left(1 + \frac{\sum_{m=n}^{\infty} \frac{R_k(m+1) - R_k(m)}{\Delta} L(0) e^{-2\sum_{p=0}^{m-1} \mu_k(p)\Delta} \Delta}{R_k(n) L(0) e^{-2\sum_{p=0}^{n-1} \mu_k(p)\Delta}}\right) \tag{S1}$$

Here we take a special two-layer numerical phantom as an example, shown in Fig. S1. We suppose the measured backscattering fraction $R_k(n)$ in each loop is equal to its theoretical value, given as,

$$R_k(n+1) - R_k(n) \approx \begin{cases} 0, n \neq N \\ 5 \times 10^{-3}, n = N \end{cases} . k \geq 1 \tag{S2}$$

Where the layer-to-layer interface is at depth of z_0, $N = \frac{z_0}{\Delta}$ and $L_0 = 1$. Under this circumstance, for the top layer, Eq. S1 could be modified as,

$$\mu(n)_{k+1} \approx \mu_0(n) \times \left(1 + \frac{\frac{R_k(m+1) - R_k(m)}{\Delta} e^{-2\sum_{p=0}^{m-1} \mu_k(p)\Delta} \Delta|_{m=N}}{R_k(n) e^{-2\sum_{p=0}^{n-1} \mu_k(p)\Delta}}\right) \quad \text{(S3)}$$

We plug Eq. S2 into it and we obtain,

$$\mu(n)_{k+1} \approx \frac{5 \times 10^{-3} \mu_0(n)}{R_k(n)} \times e^{-2\sum_{p=n}^{N-1} \mu_k(p)\Delta} + \mu_0(n), \quad \text{(S4)}$$

Where $-2\sum_{p=n+1}^{N} \mu_k(p)\Delta$ is between $(-1,0)$. We modify Eq. S4 by using Taylor first order expansion and we obtain,

$$\mu(n)_{k+1} \approx -\frac{1 \times 10^{-2} \mu_0(n)}{R_k(n)} \times \sum_{p=n}^{N-1} \mu_k(p)\Delta + \frac{5 \times 10^{-3} \mu_0(n)}{R_k(n)} + \mu_0(n). \quad \text{(S5)}$$

In this case, for the top layer of the phantom, $n \in (0, N-1)$, Eq. S5 can be modified as,

$$\begin{bmatrix} \mu_{k+1}(0) \\ \vdots \\ \mu_{k+1}(N-1) \end{bmatrix} \approx -1 \times 10^{-2}\Delta \begin{bmatrix} \frac{\mu_0(0)}{R_k(0)} & \cdots & \cdots & \frac{\mu_0(0)}{R_k(0)} \\ 0 & \frac{\mu_0(1)}{R_k(1)} & \cdots & \vdots \\ \vdots & \vdots & \ddots & \frac{\mu_0(N-1)}{R_k(N-1)} \\ 0 & 0 & & \end{bmatrix} \times \begin{bmatrix} \mu_k(0) \\ \vdots \\ \mu_k(N-1) \end{bmatrix} + 5 \times 10^{-3} \begin{bmatrix} \frac{\mu_0(0)}{R_k(0)} \\ \vdots \\ \frac{\mu_0(N-1)}{R_k(N-1)} \end{bmatrix} + \begin{bmatrix} \mu_0(0) \\ \vdots \\ \mu_0(N-1) \end{bmatrix}. \quad \text{(S6)}$$

To compute the induced norm of the iteration matrix, we suppose initial values $\mu_0(n)$ for all are equal to ideal values 0.5 mm^{-1} and $R_k(n)$ are equal to their ideal values 2×10^{-3} for all $n \in (0, N-1)$ and $k \geq 1$. Then the iteration matrix is product of a scale factor $-\frac{1\times10^{-2}\mu_0(n)\Delta}{R_k(n)}$ and an identity upper triangular matrix. $p = 1$ induced norm of this coefficient matrix is $\frac{1\times10^{-2}\mu_0(n)\Delta N}{R_k(n)}$, equal to 2.5. Recalled corollary in ref. [27], there is no global convergent solution that exists for all initial values.
